# CRISPR/Cas9-mediated editing of Δ5 and Δ6 desaturases impairs Δ8-desaturation and docosahexaenoic acid synthesis in Atlantic salmon (*Salmo salar* L.)

**DOI:** 10.1038/s41598-019-53316-w

**Published:** 2019-11-15

**Authors:** Alex K. Datsomor, Rolf E. Olsen, Nikola Zic, Angelico Madaro, Atle M. Bones, Rolf B. Edvardsen, Anna Wargelius, Per Winge

**Affiliations:** 1 Norwegian University of Science and Technology, Institute of Biology, Trondheim, 7491 Norway; 2 Institute of Marine Research, Bergen, NO-5817 Norway

**Keywords:** Fatty acids, Genetic engineering, Metabolic engineering, Transcriptomics

## Abstract

The *in vivo* functions of Atlantic salmon fatty acyl desaturases (*fads2*), *Δ6fads2-a*, *Δ6fads2-b*, *Δ6fads2-c* and *Δ5fads2* in long chain polyunsaturated fatty acid (LC-PUFA) synthesis in salmon and fish in general remains to be elucidated. Here, we investigate *in vivo* functions and *in vivo* functional redundancy of salmon *fads2* using two CRISPR-mediated partial knockout salmon, Δ6abc/5^Mt^ with mutations in *Δ6fads2-a*, *Δ6fads2-b*, *Δ6fads2-c* and *Δ5fads2*, and Δ6bc^Mt^ with mutations in *Δ6fads2-b* and Δ*6fads2-c*. F0 fish displaying high degree of gene editing (50–100%) were fed low LC-PUFA and high LC-PUFA diets, the former containing reduced levels of eicosapentaenoic (20:5n-3) and docosahexaenoic (22:6n-3) acids but higher content of linoleic (18:2n-6) and alpha-linolenic (18:3n-3) acids, and the latter containing high levels of 20:5n-3 and 22:6n-3 but reduced compositions of 18:2n-6 and 18:3n-3. The Δ6abc/5^Mt^ showed reduced 22:6n-3 levels and accumulated Δ6-desaturation substrates (18:2n-6, 18:3n-3) and Δ5-desaturation substrate (20:4n-3), demonstrating impaired 22:6n-3 synthesis compared to wildtypes (WT). Δ6bc^Mt^ showed no effect on Δ6-desaturation compared to WT, suggesting Δ6 Fads2-a as having the predominant Δ6-desaturation activity in salmon, at least in the tissues analyzed. Both Δ6abc/5^Mt^ and Δ6bc^Mt^ demonstrated significant accumulation of Δ8-desaturation substrates (20:2n-6, 20:3n-3) when fed low LC-PUFA diet. Additionally, Δ6abc/5^Mt^ demonstrated significant upregulation of the lipogenic transcription regulator, sterol regulatory element binding protein-1 (*srebp-1*) in liver and pyloric caeca under reduced dietary LC-PUFA. Our data suggest a combined effect of endogenous LC-PUFA synthesis and dietary LC-PUFA levels on *srebp-1* expression which ultimately affects LC-PUFA synthesis in salmon. Our data also suggest Δ8-desaturation activities for salmon Δ6 Fads2 enzymes.

## Introduction

**Figure 1:**
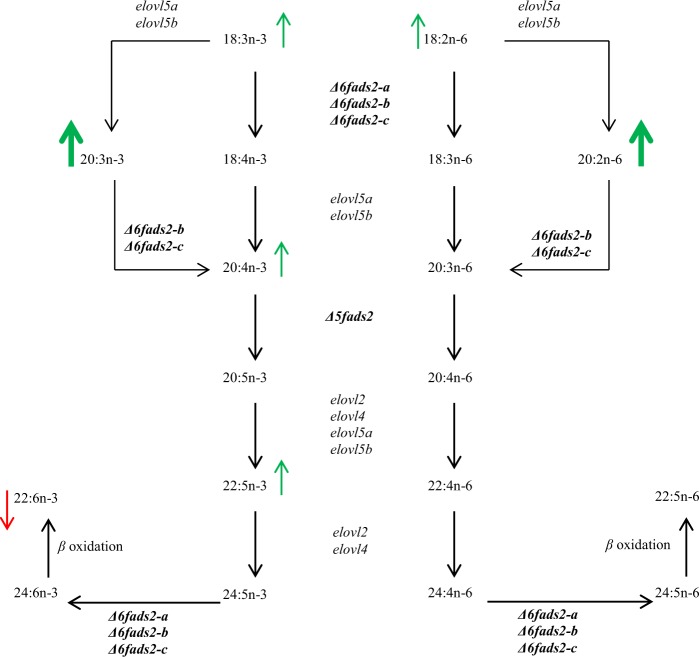
The long chain polyunsaturated fatty acid biosynthetic pathway from α-linolenic (18:3n-3) and linoleic (18:2n-6) acids. Enzymes that are mutated by CRISPR/Cas9 in the current study are in bold. The activities of the LC-PUFA synthetic enzymes in the pathway have previously been deduced *in vitro* through heterologous expression in *S. cerevisiae* of Atlantic salmon *Δ6fads2-a*^18^, *Δ6fads2-b* and *Δ6fads2-c*^17^, and of *elovl2* and *elovl5* (*elovl5a* and *elovl5b*)^16,21^. Additionally, salmon *Δ6fads2-b* and *Δ6fads2-c* have been shown to encode proteins that possess Δ8-desaturation activity *in vitro*, converting 20:3n-3 and 20:2n-6 to 20:4n-3 and 20:3n-6 respectively^13^. Elovl5 is believed to convert 18:3n-3 and 18:2n-6 to 20:3n-3 and 20:2n-6 respectively^38^. PUFAs that are affected by the CRISPR/Cas9 mutations in our study are marked with arrows. Fatty acids that have accumulated in both of our mutants, Δ6abc/5^Mt^ and Δ6bc^Mt^ are marked with bold green arrows and those that accumulated mostly in only Δ6abc/5^Mt^ are marked with narrow green arrows. LC-PUFA that has shown reduced percentage composition in Δ6abc/5^Mt^ is marked with a red arrow.

The health benefits of fish oil particularly eicosapentaenoic acid (20:5n-3) and docosahexaenoic acid (22:6n-3) have been demonstrated by many studies. These omega-3 long chain (≥C_20_) polyunsaturated fatty acids (n-3 LC-PUFAs) are known to reduce incidences of cardiovascular diseases, inflammatory disorders and neurological pathologies in humans^1–6^. Farmed fish, including Atlantic salmon (*Salmo salar* L.) currently provides an increasing proportion of n-3 LC-PUFAs in human diet^7–9^. Consequently, there has been interest in understanding endogenous synthesis and regulation of LC-PUFAs in Atlantic salmon, which is the most farmed species of salmonids^9,10^. The LC-PUFA biosynthetic pathway in Atlantic salmon is similar to that of majority of other vertebrates^7,9,11^ (Fig. 1). Biosynthesis of LC-PUFAs in vertebrates requires sequential desaturation and elongation of the C_18_ PUFAs, α-linolenic acid (18:3n-3) and linoleic acid (18:2n-6). Synthesis of 20:5n-3 is achieved via Δ6-desaturation of 18:3n-3 to 18:4n-3, which is elongated to 20:4n-3 followed by Δ5-desaturation. Synthesis of arachidonic acid (20:4n-6) requires the same enzymes and involves Δ6-desaturation of 18:2n-6 to 18:3n-6 that is elongated to 20:3n-6 followed by Δ5-desaturation^12^. Alternatively, 18:3n-3 and 18:2n-6 may be elongated to eicosatrienoic acid (20:3n-3) and eicosadienoic acid (20:2n-6) respectively, followed by Δ8-desaturation to 20:4n-3 and 20:3n-6^13^, which are respectively converted to 20:5n-3 and 20:4n-6 via Δ5-desaturation. Biosynthesis of 22:6n-3 requires two elongation steps from 20:5n-3, a second Δ6-desaturation and a chain-shortening step by peroxisomal β-oxidation in the so called “Sprecher pathway”^14^. 22:6n-3 may be directly synthesized through Δ4-desaturation of docosapentaenoic acid (22:5n-3)^15^. However, the latter pathway may not exist in Atlantic salmon.

The capacity for LC-PUFA synthesis in any species depends on complementary activities of fatty acyl desaturases (Fads2) and elongases of very long chain fatty acid (Elovls). Multiple Atlantic salmon Δ6 *fads2* (*Δ6fads2-a*, *Δ6fads2-b* and *Δ6fads2-c*) and *Δ5fads2* genes have been cloned and functionally characterized through heterologous expression in the yeast *Saccharomyces cerevisiae*^16–18^. The salmon Δ6 *fads2* genes encode proteins that predominantly possess Δ6-desaturation activity towards 18:3n-3,18:2n-6, 24:4n-6 and 24:5n-3^17–19^, while *Δ5fads2* gene encodes an enzyme with predominant Δ5-desaturation activity towards 20:4n-3 and 20:3n-6 with some Δ6-desaturation of 18:3n-3,18:2n-6, 24:4n-6 and 24:5n-3^16,19^. Additionally, the salmon *elovl* genes, *elovl2*, *elovl4*, *elovl5a* and *elovl5b* have been cloned and functionally characterized through *in vitro* studies in *S. cerevisiae*^16,20,21^. Salmon *elovl2* and *elovl4* encode proteins that efficiently elongate C_20_ and C_22_ LC-PUFAs^20,21^, whereas *elovl5a* and *elovl5b* encode enzymes that elongate C_18_ and C_20_ PUFAs with marginal activities towards C_22_ LC-PUFAs^16,21^. The Atlantic salmon LC-PUFA pathway responds to dietary PUFA compositions. Significant upregulation of *Δ6fads2-a* and *Δ6fads2-b*^17^ as well as *elovl5b* and *elovl2*^21^ genes was observed in salmon fed diets rich in 18:3n-3 and 18:2n-6 but devoid of 20:5n-3 and 22:6n-3. Similarly, a diet high in 18:3n-3 and 18:2n-6 increases enzymatic activity of fatty acyl desaturases and elongases for elongation of very long chain fatty acid in Atlantic salmon, compared to a diet high in 20:5n-3 and 22:6n-3^22^. Dietary fatty acids control lipogenic gene expression through direct or indirect interaction with transcription regulators, for example liver-x-receptor-alpha (Lxr-α) and sterol regulatory element binding protein-1 (Srebp-1)^23,24^. An Lxr-α-response element has been identified within the promoter of Srebp-1c and was shown to be the primary site mediating LC-PUFA-dependent regulation of Srebp-1c in rat^23^. In this study, 22:6n-3-mediated repression of Srebp-1c expression was found to be Lxr-α-dependent in rat hepatocyte assays^23^. Indeed, in assessing LC-PUFA biosynthetic capacity of Atlantic salmon and other salmonids, such as rainbow trout, Artic charr and brown trout, numerous *in vitro* studies involving heterologous expression^13,16,17,20,21^ and fatty acid desaturation/elongation assays in both hepatocytes and enterocytes^22,25,26^ have been performed. To broaden and provide detailed insight into the *in vivo* functions of genes encoding salmon LC-PUFA biosynthetic enzymes, as well as understand nutritional and transcriptional regulation of LC-PUFA biosynthesis, *in vivo* functional studies are required.

Programmable DNA endonucleases have been used for *in vivo* functional studies in many animal models over the past years. Clustered regularly interspaced short palindromic repeat (CRISPR)/CRISPR-associated (Cas) system^27–29^ has been proven to be an efficient and cost-effective genome editing tool^30^. CRISPR-induced frame shift insertions and deletions (indels) create loss-of-gene function by altering protein-coding region or by premature termination codons (PTCs) that produce truncated proteins as well as signal the nonsense-mediated mRNA decay (NMD) pathway that recognizes and degrade aberrant mRNAs^31^. CRISPR/Cas9 has been successfully used to induce biallelic mutations in the F0 of zebrafish^32^ and Atlantic salmon^33,34^ allowing for phenotypic analysis directly in the F0 animals. Taking advantage of previous heterologous studies in *S. cerevisiae* reporting Δ8-desaturation activities for salmon Δ6 Fads2-b and Δ6 Fads2-c^13^, together with data from initial *in vitro* studies ranking Δ6-desaturation activities of the salmon Δ6 desaturases as Δ6 Fads2-a > Δ6 Fads2-b > Δ6 Fads2-c, we generated partial salmon knockouts in two different combinations (Δ6abc/5^Mt^ and Δ6bc^Mt^) using established CRISPR/Cas9 protocols for Atlantic salmon^33,34^. Δ6bc^Mt^ provided insights into *in vivo* Δ6-desaturation capacity as well as *in vivo* Δ8-desturation activities of the salmon Δ6 Fads2-b and Δ6 Fads2-c. Comparison between Δ6abc/5^Mt^ and Δ6bc^Mt^ provided some understanding of the predominant Δ6-desaturation roles of Δ6 Fads2-a *in vivo* compared to Δ6 Fads2-b and Δ6 Fads2-c. Due to the long generation time of salmon, F0 fish with high percentage of targeted mutations in genes of interest were used in the functional analysis. We could thereby demonstrate that 20:4n-3 and 18:3n-3/18:2n-6 is the main *in vivo* substrate of salmon Δ5 Fads2 and Δ6 Fads2, respectively. Additionally, we show that the salmon Δ6 Fads2 possess Δ8-desaturation activities towards 20:3n-3 and 20:2n-6 *in vivo*. We also observed that *Δ6fads2-a* has a more dominant role compared to its paralogous genes *Δ6fads2-b* and *Δ6fads2-*c in 18:3n-3 and 18:2n-6 desaturation under the given conditions and in tissue types analyzed. Our data further suggest Srebp-1 as a major transcription regulator of salmon LC-PUFA biosynthesis and show that the status of endogenous LC-PUFA synthesis as well as dietary LC-PUFA composition control expression of *srebp-1*.

## Results

### Generation of Δ6abc/5^Mt^ and Δ6bc^Mt^, confirmation of CRISPR/Cas9-induced mutations and Growth performance

Two groups of CRISPR/Cas9-mutated salmon, Δ6abc/5^Mt^ and Δ6bc^Mt^ were generated as previously described in Atlantic salmon^33^. To provide a suitable visual screening of the knockouts from WT, the *slc45a2* gene involved in melanin synthesis was simultaneously mutated with the *fads2* genes. CRISPR/Cas9-induced indels in the *fads2* genes were highly correlated with the albino phenotype (Supplemental Table 1) in line with previously reported results^34,35^. Mutations were confirmed through direct sequencing of PCR fragments isolated from gels and sub-cloned PCR products flanking regions around each target site. CRISPR/Cas9-induced mutations were detected as scrambled peaks from target sites in DNA sequencing chromatograms (Supplemental Fig. 1A–E). Mutations were observed in all targeted *fads2* genes, *Δ6fads2-a*, *Δ6fads2-b*, *Δ6fads2-c* and *Δ5fads2* in individuals of Δ6abc/5^Mt^ group (Supplemental Fig. 1A–D) and in PCR products obtained from co-amplification of *Δ6fads2-b* and *Δ6fads2-c* in Δ6bc^Mt^ individuals (Supplemental Fig. 1E). The results were validated by sequencing of sub-cloned PCR-products. The Δ6abc/5^Mt^ group has predominantly 5 bp deletions at CRISPR-target sites regardless of the gene targeted. Other different types of indels including deletions and insertions were observed. The predominant indel in Δ6bc^Mt^ group is a 4 bp deletion in addition to other different types of insertions and deletions. To study the impact of *fads2* gene knockout and dietary LC-PUFA levels on endogenous LC-PUFA biosynthesis, fish were fed low LC-PUFA and high LC-PUFA diets (Supplemental Table 2). Prior to the feeding trial, fish were fed a standard commercial diet (Supplemental Table 2) shortly after hatching, where Δ6bc^Mt^ and Δ6abc/5^Mt^ were notably similar in size with estimated average weight of 49 g but smaller than WT with approximate weight of 85 g (Table 1). Consequently, fish were fed 22:6n-3 (DHA)-rich diets (Supplemental Table 2) to enhance growth. Of all three groups of experimental fish, Δ6abc/5^Mt^ displayed reduced average weight regardless of dietary treatment with DHA-rich, low LC-PUFA or high LC-PUFA diet compared to Δ6bc^Mt^ and WT (Table 1). Surprisingly, average weights of all experimental groups seem to be higher when fed low LC-PUFA diet compared to fish fed high dietary LC-PUFA, with Δ6bc^Mt^ showing the highest average weight (Table 1).

**Table 1:** Weights and lengths of experimental fish fed different dietary regimens.

	Weight (g)	Length (cm)	Number of fish	Dietary treatment	Feeding duration (days)
WT^**^	≈85	—	72	Standard diet	227
Δ6abc/5^Mt**^/Δ6bc^Mt^	≈49	—	72 each		
Δ6abc/5^Mt^	85 ± 25^**a**^	19 ± 2^**a**^	36	DHA-rich diets	110
Δ6bc^Mt^	104 ± 25^**b**^	20 ± 2^**b**^	36		
WT	176 ± 34^**c**^	24 ± 2^**c**^	36		
Δ6abc/5^Mt^	203 ± 51^**ac**^	27 ± 2	6	Low LC-PUFA diet	54
Δ6bc^Mt^	281 ± 52^**b**^	30 ± 2	6		
WT	250 ± 62^**ab**^	30 ± 5	6		
Δ6abc/5^Mt^	171 ± 36^**c**^	26 ± 1	6	High LC-PUFA diet	54
Δ6bc^Mt^	191 ± 69^**ac**^	27 ± 3	6		
WT	241 ± 47^**ab**^	29 ± 1	6		

Shortly after hatching fish were fed a standard commercial diet followed by 22:6n-3 (DHA)-rich diets to enhance growth. Fish were subsequently fed two experimental diets, low LC-PUFA and high LC-PUFA diets. **Weights of WT and Δ6abc/5^Mt^ were estimated, as experimental fish were weighed together in two groups or categories as Pit-tagged (Δ6bc^Mt^) or untagged (WT + Δ6abc/5^Mt^). At the time of switching from standard diet to 22:6n-3 (DHA)-rich diets, we did not weigh Δ6abc/5^Mt^ group separately but they were observed to have similar size as Δ6bc^Mt^ compared to WT. Weights and lengths of fish fed low and high LC-PUFA diets were analyzed by two-way ANOVA using dietary treatment and strain or genotype as experimental factors, followed by multiple comparisons of the means using Tukey HSD post-hoc test. Weights and lengths of fish fed DHA-rich diets were analyzed by one-way ANOVA using strain as experimental factor, followed by Tukey HSD post-hoc test. Different superscripts indicates statistical difference, p < 0.05.

### Δ6abc/5^Mt^ shows impaired synthesis of 22:6n-3

**Figure 2:**
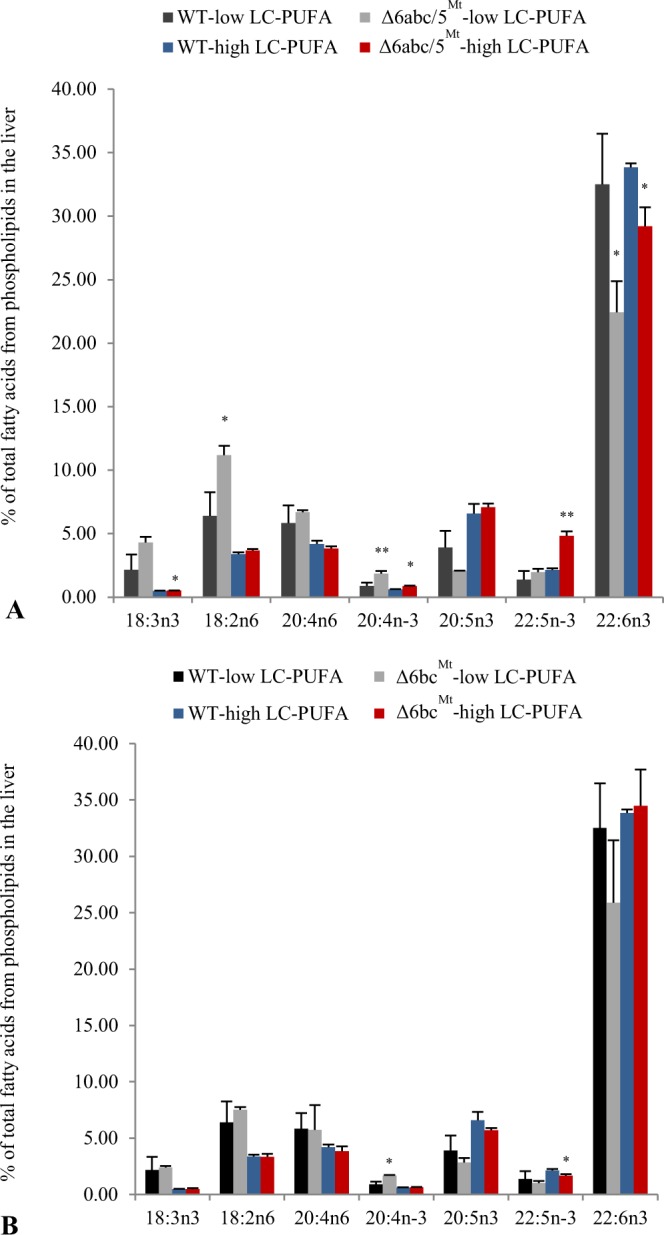
Δ6abc/5^Mt^ shows impaired liver phospholipid 22:6n-3 synthesis, displayed as reduced phospholipid 22:6n-3 levels and accumulation of 18:3n-3, 18:2n-6, 20:4n-3 and 22:5n-3 compared to WT (**A**). Δ6bc^Mt^ shows no significant effect on Δ6-desaturation substrates (18:3n-3 and 18:2n-6) and 22:6n-3 synthesis compared to WT, however, an unexpected accumulation of 20:4n-3 was observed when Δ6bc^Mt^ were fed low LC-PUFA diet (**B**). The wildtypes (WT), Δ6abc/5^Mt^ and Δ6bc^Mt^ were fed low LC-PUFA and high LC-PUFA diets for 54 days. For easy comparison, the same WT data is presented both in A and B. Phospholipids were separated on high performance thin layer chromatography silica gel 60 plates. Fatty acid methyl esters (FAMEs) were prepared by acid-catalyzed transesterification and quantified by gas chromatography coupled with mass spectroscopy. Results are shown as mean ± standard deviation of liver samples from 3 fishes. Statistical differences between WT and CRISPR-mutated fish were determined using two-tailed t-test with unequal variance and are denoted as asterisks (*p ≤ 0.05 and **p < 0.01).

To understand *in vivo* functions and evaluate possible *in vivo* functional redundancy of the desaturases, CRISPR-mutated salmon from groups, Δ6abc/5^Mt^ and Δ6bc^Mt^ were fed low and high LC-PUFA diets for 54 days. Liver phospholipid PUFA composition in all three fish groups, especially of 18:2n-6 and 18:3n-3 showed a positive correlation with the dietary contents after the 54 days of feeding (Fig. 2A,B). Additionally, we observed an accumulation of liver phospholipid 18:3n-3, 18:2n-6, 20:4n-3 and 22:5n-3 in Δ6abc/5^Mt^ compared with WTs (Fig. 2A). This was accompanied by a reduction in the levels of liver phospholipid 22:6n-3. Similar observation was made from white muscle phospholipids of Δ6abc/5^Mt^ group fed low LC-PUFA diet even though changes were subtle especially when fed a high LC-PUFA diet (Supplemental Fig. 2). In general, there were no significant changes in the levels of liver phospholipid 18:3n-3 and 18:2n-6 in the Δ6bc^Mt^ group compared to WT (Fig. 2B). However, a significant but unexpected accumulation of 20:4n-3 was observed in the liver of Δ6bc^Mt^ group fed a low LC-PUFA diet (Fig. 2B).

### Δ6abc/5^Mt^ and Δ6bc^Mt^ demonstrate accumulation of Δ8-desaturation substrates

**Figure 3:**
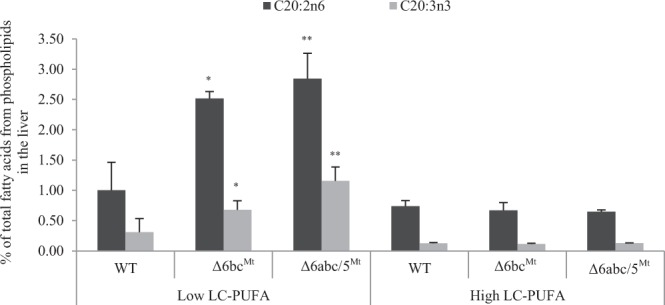
Δ6abc/5^Mt^ and Δ6bc^Mt^ accumulate 20:2n-6 and 20:3n-3 phospholipids in the liver when fed low LC-PUFA diet rich in 18:2n-6 and 18:3n-3. The wildtypes (WT), Δ6abc/5^Mt^ and Δ6bc^Mt^ were fed low LC-PUFA and high LC-PUFA diets for 54 days. Phospholipids were separated on high performance thin layer chromatography silica gel 60 plates. Fatty acid methyl esters (FAMEs) were prepared by acid-catalyzed transesterification and quantified by gas chromatography coupled with mass spectroscopy. Results for tissue fatty acid composition are shown as mean ± standard deviation of liver samples from 3 fishes. Statistical differences between WT and CRISPR-mutated fish were determined using two-tailed t-test with unequal variance and are denoted as asterisks (*p ≤ 0.05 and **p < 0.01).

Analysis of PUFA composition in liver phospholipids revealed a clear accumulation of precursors for Δ8-desaturation (20:3n-3 and 20:2n-6) in Δ6abc/5^Mt^ and Δ6bc^Mt^ compared with the WTs (Fig. 3). This was only obvious in fish that were fed low LC-PUFA diet containing high levels of 18:3n-3 and 18:2n-6 compared to the high LC-PUFA diet (Fig. 3). Furthermore, the levels of liver phospholipid 20:3n-3 and 20:2n-6 correlate well with dietary levels of the C_18_ precursors (Fig. 3 and Supplemental Table 2), with fish fed low LC-PUFA diet generally having higher levels of the C_20_ Δ8-desaturation substrates compared with fish fed high LC-PUFA diet. There was no change in liver phospholipid 20:3n-3 and 20:2n-6 in Δ6abc/5^Mt^ and Δ6bc^Mt^ that were fed high LC-PUFA diet (Fig. 3). Similar observation was made from white muscle phospholipid of Δ6abc/5^Mt^ and Δ6bc^Mt^ fish compared with the WTs (Supplemental Fig. 3A). Additionally, accumulation of the C_20_ Δ8-desaturation substrates was observed in white muscle triacylglycerol (TAG) of Δ6abc/5^Mt^ and Δ6bc^Mt^ that were fed low LC-PUFA diet (Supplemental Fig. 3B).

### Δ6abc/5^Mt^ shows impaired liver Δ8-desaturation of ^14^C-20:3n-3 and n-3 PUFA synthesis from ^14^C-18:3n-3

**Figure 4:**
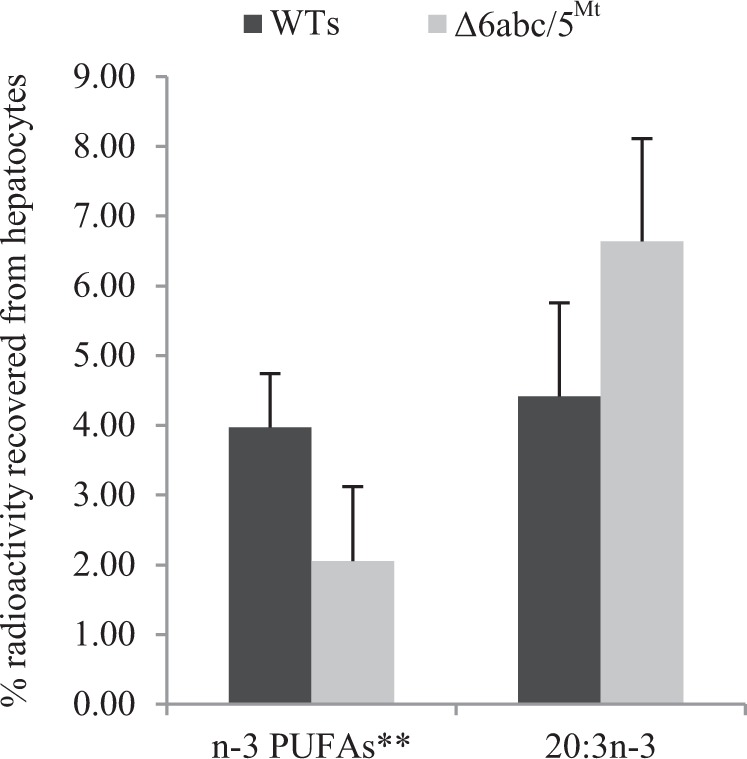
The degree of ^14^C-18:3n-3 desaturation in hepatocytes shown as percentage of radioactivity recovered in 20:3n-3, a substrate for Δ8-desaturation, and the overall impact of CRISPR-induced mutations in Δ6abc/5^Mt^ on n-3 PUFA synthesis shown as percentage radioactivity recovered in n-3 PUFAs** (18:4n-3, 20:4n-3, 20:5n-3 and 22:5n-3) determined individually but pooled together. Approximately 90% of radioactivity was recovered from ^14^C-18:3n-3 in all samples. Data represent mean ± standard deviation of 3 and 2 samples from WT and the Δ6abc/5^Mt^ respectively.

To validate our observations, hepatocytes from WTs and Δ6abc/5^Mt^ salmon that were fed low LC-PUFA diet were analyzed for the ability to convert radiolabeled 18:3n-3 to n-3 PUFAs. Hepatocytes were incubated with ^14^C-18:3n-3 and the percentages of radioactivity recovered from 18:4n-3, 20:4n-3, 20:5n-3 and 22:5n-3 individually determined and pooled together. The percentage of ^14^C-20:3n-3, a substrate for Δ8-desaturation was also determined. Desaturation/elongation capacity of hepatocytes from Δ6bc^Mt^ could not be assayed as a result of reduced radioactivity signal strength from samples, most likely due to limiting starting materials. Hepatocytes from Δ6abc/5^Mt^ salmon showed a clear accumulation of ^14^C-20:3n-3 and reduced percentage radioactivity recovered as the PUFAs (18:4n-3, 20:4n-3, 20:5n-3 and 22:5n-3) compared to WTs (Fig. 4). However, these results were not statistically significant. Furthermore, it appears 22:6n-3 being relatively longer and with higher degree of unsaturation could not migrate from the origin and was not quantified with certainty, this was unexpected as the recommended experimental protocols were followed.

### Δ6abc/5^Mt^ demonstrates low dietary LC-PUFA-induced mRNA expression of *srebp-1*

**Figure 5:**
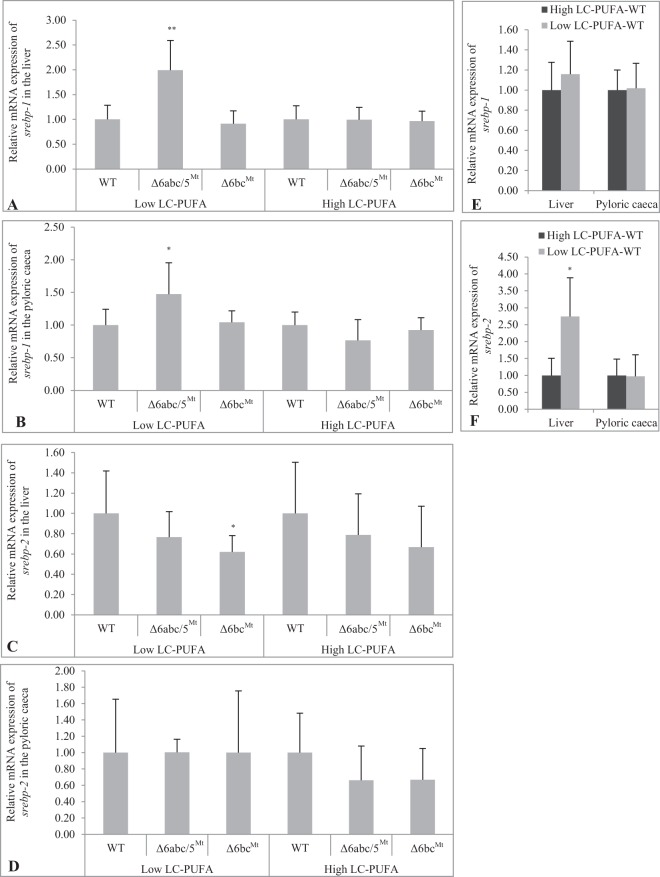
Δ6abc/5^Mt^ showed low dietary LC-PUFA-induced expression of sterol regulatory element binding protein-1 (*srebp-1*) in the liver (**A**) and pyloric caeca (**B**). The Δ6abc/5^Mt^ showed no significant effect on the expression of *srebp-2* (C and D), however, mRNA expression of *srebp-2* was downregulated in the liver of Δ6bc^Mt^ when fed low LC-PUFA diet (**C**).The mRNA expression of *srebp-1* and *srebp-2* in CRISPR-mutated fish was determined relative to wildtypes (WT), with WT set to 1. The impact of the two diets on the expression of *srebp-1* and *srebp-2* was determined by measuring mRNA expression in WT fed low LC-PUFA diet relative to WT fed high dietary LC-PUFA, with WT fed high LC-PUFA diet set to 1. Differences in dietary LC-PUFA showed no significant effect on mRNA expression of *srebp-1* (**E**). However, WT fed low LC-PUFA diet showed upregulated mRNA expression of *srebp-2* in the liver (**F**). WT, Δ6abc/5^Mt^ and Δ6bc^Mt^ were fed low LC-PUFA and high LC-PUFA diets for 54 days. All qPCR data were analyzed using qBase^+^^51^ which determined statistical differences between WT and CRISPR-mutated fish and between WT under the two dietary regimens by unpaired Mann-Whitney test with two-sided significance. Data are presented as means ± confidence interval with N = 5 per dietary treatment. Normalization was performed using elongation factor 1α-b (*ef1α-b*). Statistical differences between WT and CRISPR-mutated fish are denoted as asterisks (*p < 0.05, **p < 0.01).

The mRNA expression levels of the lipogenic transcription regulators; *srebp-1* and *srebp-2* in Δ6abc/5^Mt^ and Δ6bc^Mt^ groups, were measured in liver and intestine (pyloric caeca) using RT-qPCR. We observed significant (p < 0.05) upregulation of *srebp-1* in the liver and pyloric caeca of Δ6abc/5^Mt^ salmon fed low LC-PUFA diet (Fig. 5A,B). Upregulation of *srebp-1* was higher in the liver (≈2 folds) compared to pyloric caeca (≈1.5 folds). To validate the observed increased expression of *srebp-1*, we measured liver and intestinal (pyloric caeca) mRNA expression levels of fatty acid synthase-a (*fas-a*) and *fas-b*, which are key downstream target genes of Srebp-1^36^. Liver *fas-a* and *fas-b* were upregulated in Δ6abc/5^Mt^ fed a low LC-PUFA diet (Supplemental Fig. 4A,B), although not statistically significantly. The mRNA expression levels of intestinal *fas-a* and *fas-b* remain unchanged (data not shown). Additionally, no upregulation of *srebp-1* was observed in Δ6abc/5^Mt^ salmon fed a high LC-PUFA diet or in Δ6bc^Mt^ salmon fed either low or high LC-PUFA diet (Fig. 5A,B). Δ6abc/5^Mt^ showed no significant change in the expression of *srebp-2* (Fig. 5C,D). However, a significant reduction in the mRNA levels of *srebp-2* was observed in the liver of Δ6bc^Mt^ when fed low LC-PUFA diet (Fig. 5C). The impact of the two dietary regimens on the expression levels of *srebp-1* and *srebp-2* was determined by comparing expression levels in WTs fed low LC-PUFA diet relative to WTs fed high dietary LC-PUFA. The two diets had no significant effect on the expression of *srebp-1* (Fig. 5E). However, a significant increase in the expression of *srebp-2* was observed in the liver of WTs fed low LC-PUFA diet (Fig. 5F).

### Δ6abc/5^Mt^ and Δ6bc^Mt^ mutants show reduced expression of CRISPR/Cas9-targeted genes

RT-qPCR analysis of CRISPR-targeted genes showed reduced mRNA expression of *Δ6fads2-a*, *Δ6fads2-b* and *Δ5fads2* in Δ6abc/5^Mt^, *Δ6fads2-b* and *Δ6fads2-c* in Δ6bc^Mt^ in both liver and pyloric caeca (Table 2). Unexpectedly, *Δ5fads2* appears to be significantly downregulated in liver of Δ6bc^Mt^. Expression of *Δ5fads2* was also slightly downregulated in pyloric caeca of Δ6bc^Mt^, but this was not statistically significant. Reduced mRNA expression of CRISPR-target genes appears to be relatively stronger in Δ6abc/5^Mt^ fed on high LC-PUFA diet than on low LC-PUFA diet and appears to correlate well with mRNA expression of *srebp-1*. This observation was made both in the liver and pyloric caeca.

**Table 2:** Relative expression of CRISPR-targeted genes in Δ6abc/5^Mt^ and Δ6bc^Mt^ fed low and high LC-PUFA diets for 54 days.

Diet fed over 54 days	Gene	Relative expression in liver			Relative expression in pyloric caeca		
		WT	Δ6bc^Mt^	Δ6abc/5^Mt^	WT	Δ6bc^Mt^	Δ6abc/5^Mt^
Low LC-PUFA	*Δ6fads2-a*	1.00 ± 0.58	0.63 ± 0.17	**0.33 **± 0.32*	1.00 ± 1.47	1.14 ± 0.30	0.32 ± 0.63
	*Δ6fads2-b*	1.00 ± 0.38	**0.26 **± 0.16**	0.80 ± 0.99	1.00 ± 1.55	0.47 ± 0.38	1.20 ± 2.14
	*Δ6fads2-c*	1.00 ± 0.53	0.52 ± 0.36	1.89 ± 2.21	1.00 ± 0.18	0.81 ± 0.42	0.66 ± 0.36
	*Δ5fads2*	1.00 ± 0.41	**0.37 **± 0.09**	**0.17 **± 0.12*	1.00 ± 2.66	1.22 ± 0.48	0.33 ± 0.55
High LC-PUFA	*Δ6fads2-a*	1.00 ± 0.94	0.67 ± 0.09	**0.18 **± 0.11**	1.00 ± 0.17	0.94 ± 0.47	**0.15 **± 0.27**
	*Δ6fads2-b*	1.00 ± 0.36	**0.50 **± 0.15*	**0.41 **± 0.27*	1.00 ± 1.28	0.34 ± 0.22*	**0.30 **± 0.33*
	*Δ6fads2-c*	1.00 ± 0.47	**0.54 **± 0.08**	0.91 ± 1.06	1.00 ± 0.37	**0.53 **± 0.20*	0.91 ± 0.95
	*Δ5fads2*	1.00 ± 0.44	**0.50 **± 0.12**	**0.07 **± 0.07**	1.00 ± 0.28	0.69 ± 0.60	**0.06 **± 0.09**

The mRNA expression of CRISPR-targeted genes in Δ6abc/5^Mt^ and Δ6bc^Mt^ was determined relative to wildtypes (WT), with WT set to 1. Data are presented as means ± confidence interval with N = 5 per dietary treatment. Statistical differences between WT and CRISPR-mutated fish are denoted as asterisks and are in bold (*p ≤ 0.05, **p < 0.01).

## Discussion

The current study sought to understand *in vivo* functions of Atlantic salmon desaturases and to evaluate their levels of *in vivo* functional redundancy in LC-PUFA biosynthesis using two groups of CRISPR-mutated salmon, Δ6abc/5^Mt^ with mutated *Δ6fads2-a*, *Δ6fads2-b*, *Δ6fads2-c* and *Δ5fads2* genes and Δ6bc^Mt^ where only *Δ6fads2-b* and *Δ6fads2-c* were mutated. Additionally, nutritional and transcriptional regulation of Atlantic salmon LC-PUFA biosynthesis was investigated. Our data from Δ6abc/5^Mt^ and Δ6bc^Mt^ salmon indicate that Δ6 Fads2-a is responsible for the largest proportion of Δ6-desaturation activity in liver, and that salmon Δ6 Fads2 have Δ8-desaturation activity towards 20:2n-6 and 20:3n-3 *in vivo*. Furthermore, we have shown that both the status of endogenous LC-PUFA synthesis and dietary LC-PUFA levels influence the expression of *srebp-1*, a major transcription regulator that controls the expression of lipogenic enzymes involved in LC-PUFA synthesis^37^.

The Δ6abc/5^Mt^ salmon demonstrated impaired synthesis of 22:6n-3, displayed as reduced levels of 22:6n-3 and accumulation of Δ6-desaturation substrates (18:2n-6 and 18:3n-3) as well as 20:4n-3 which is a substrate for Δ5-desaturation. Additionally, accumulation of 22:5n-3, which is not a direct substrate of salmon Δ6 Fads2 and Δ5 Fads2 but a precursor upstream of the final Δ6-desaturation (Fig. 1) required for 22:6n-3 synthesis was observed. We cannot exclude some contributions of *Δ5fads2* knockout on the accumulation of 18:3n-3 and 18:2n-6, as Atlantic salmon Δ5 Fads2 maintains some Δ6-desaturation activities when heterologously expressed in *S. cerevisiae*^16,19^. However, this is marginal as salmon Δ5 Fads2 could only convert 0.4 and 0.6% of 18:2n-6 and 18:3n-3, respectively in *S. cerevisiae*^16^. On the other hand, salmon Δ5 Fads2 showed 1.4 and 6.4% conversion towards the C_24_ Δ6-desaturation substrates, 24:4n-6 and 24:5n-3, respectively when expressed in *S. cerevisiae*^19^. The impact of CRISPR-induced mutations in Δ6abc/5^Mt^ on tissue LC-PUFAs is influenced by dietary PUFA composition. Accordingly, accumulation of Δ6-desaturation substrates were observed in Δ6abc/5^Mt^ that were fed low LC-PUFA diet which contains high levels of the C_18_ precursors compared to high LC-PUFA diet. Notably, Δ6abc/5^Mt^ demonstrated reduced average weight compared to Δ6bc^Mt^ and WT, regardless of dietary treatment with DHA-rich diets or low LC-PUFA and high LC-PUFA diets. This probably suggests that impaired LC-PUFA biosynthesis in Δ6abc/5^Mt^ compared to Δ6bc^Mt^ may have to some extent affected growth; however, this requires further studies with specially designed growth trials to ascertain. On the other hand, Δ6bc^Mt^ had the highest average weight compared to other fish groups when fed low LC-PUFA diet, this was interesting but unexpected, and the reason for this observation is unclear. Surprisingly, all experimental fish groups showed relatively higher average weight when fed low LC-PUFA diet compared to high LC-PUFA feed. Although the higher total PUFA content in low LC-PUFA diet could be a contributory factor, further investigations with higher sample size are needed to ascertain these observations. The impact of CRISPR-induced mutations in Δ6bc^Mt^ on LC-PUFA synthesis was rather subtle. Thus, while it is expected that knockout of *Δ6fads2-b* and *Δ6fads2-c* significantly reduce Δ6-desaturation of 18:2n-6 and 18:3n-3, the Δ6bc^Mt^ generally demonstrated no or little accumulation of the C_18_ precursors compared to WT. This suggests that *Δ6fads2-a* encodes the main Δ6-desaturation enzyme in the salmon tissues analyzed in our study, particularly liver and to some extent white muscle. This result is supported by findings obtained from heterologous studies in *S*. *cerevisiae*, where Δ6-desaturation activities of salmon Δ6 Fads2 enzymes are ranked as Δ6 Fads2-a > Δ6 Fads2-b > Δ6 Fads2-c^16,17^.

Interestingly, accumulation of the Δ8-desaturation substrates was observed in both Δ6abc/5^Mt^ and Δ6bc^Mt^ fed low LC-PUFA diet, which contains very low levels of the C_20_ Δ8 precursors. It is probable that the observed Δ8-desaturation substrates are products of Elovl5-mediated elongation of 18:2n-6 and 18:3n-3^38^. In support of our reasoning, tissue composition of phospholipid 20:3n-3 and 20:2n-6 of fish fed low LC-PUFA diet correlates well with dietary levels of the C_18_ precursors. Generally, there were no significant differences in the level of accumulation of the Δ8-desaturation substrates between Δ6abc/5^Mt^ and Δ6bc^Mt^, suggesting that salmon Δ6 Fads2-b and Δ6 Fads2-c either individually or collectively have higher Δ8-desaturation activity than Δ6 Fads2-a. Further studies are required to confirm the Δ8-desaturation capabilities of Δ6 Fads2-a as Δ8-desaturation activity has only been investigated for salmon Δ6 Fads2-b and Δ6 Fads2-c^13^. Additionally, there were no changes in the levels of the Δ8-desaturation substrates in Δ6abc/5^Mt^ and Δ6bc^Mt^ when fed high dietary LC-PUFAs. This suggests that the Δ8 biosynthetic pathway (C_18_ elongation → C_20_ Δ8-desaturation → C_20_ Δ5-desaturation) may be activated by high dietary levels of 18:3n-3 and 18:2n-6, and functions together with the Δ6-pathway (C_18_ Δ6-desaturation → C_18_ elongation → C_20_ Δ5-desaturation) to enhance conversion of C_18_ precursors to essential LC-PUFAs under limiting conditions.

As expected, our gene expression data revealed reduced mRNA levels of CRISPR-target genes including *Δ6fads2-a*, *Δ6fads2-b* and *Δ5fads2* in Δ6abc/5^Mt^ and *Δ6fads2-b* and *Δ6fads2-c* in Δ6bc^Mt^. On the other hand, *Δ6fads2-c* expression levels in Δ6abc/5^Mt^ showed no significant reduction. Additionally, an unexpected downregulation of *Δ5fads2* was observed in Δ6bc^Mt^, which is significant in liver but not in pyloric caeca. This was surprising as the reduced expression in Δ6bc^Mt^ is mostly limited to the liver, compared to Δ6abc/5^Mt^ where the targeted *Δ5fads2* is significantly downregulated both in liver and pyloric caeca when fed high LC-PUFA diet. Even though the CRISPR-construct targeting *Δ6fads2-b* and *Δ6fads2-c* in Δ6bc^Mt^ was pre-validated *in silico* to ensure high on-target specificity, we cannot exclude possibilities of off-target effects. We attempted to assess possible off-target indels in liver transcripts using RNAseq data (data to be published in a separate manuscript), even though no off-target gene editing was observed, the data was not sufficient to prove with certainty absence of off-target indels. Nonsense-mediated mRNA decay (NMD), a translation-dependent mRNA surveillance pathway, has been shown to recognize and eliminate mRNAs containing premature termination codons (PTCs)^31^. Based on our sequence data from direct PCR fragments and sub-cloned PCR products, the Δ6abc/5^Mt^ and Δ6bc^Mt^ showed out-of-frame mutations in the respective target genes, which would normally generate PTCs. Reduced mRNA levels of the *fads2* genes in Δ6abc/5^Mt^ appears to correlate well with the expression of *srebp-1* suggesting the *Δ6 fads2* and *Δ5fads2* genes are targets of Srebp-1, which is consistent with findings in the Atlantic salmon head kidney cells (SHK-1)^37^. Notably, the reduction in mRNA levels of the *fads2* genes in Δ6abc/5^Mt^ appears to be influenced by dietary LC-PUFAs, with reduction in mRNA expression being stronger under high LC-PUFA diet. These results suggest a LC-PUFA-dependent regulation of the salmon *fads2* genes probably in a Srebp-1-dependent fashion (Fig. 5A,B). Previous *in vitro* studies in Atlantic salmon SHK-1 cells demonstrated regulation of Srebp-1 and LC-PUFA biosynthetic enzymes by dietary 20:5n-3 and 22:6n-3^37^. In the present study, the impaired synthesis of 22:6n-3 in Δ6abc/5^Mt^ appears to significantly upregulate *srebp-1* in the liver and pyloric caeca under low LC-PUFA diet, which is consistent with results reported from studies in mice^36^. Our results show higher upregulation of *srebp-1* in the liver (≈2 folds) than in the pyloric caeca (≈1.5 folds), which is in line with the fact that liver is the main metabolic organ controlling systemic lipid metabolism^39^. Δ6abc/5^Mt^ salmon fed high LC-PUFA diet, which contains relatively high levels of 20:5n-3 and 22:6n-3, showed no upregulation of liver or intestinal *srebp-1*, despite significant reduction of 22:6n-3 in the liver. This suggests a feedback-inhibitory effect of dietary LC-PUFAs on *srebp-1* expression, in line with results previously reported in SHK-1 cells by Minghetti *et al*.^37^. While it is compelling to reason that increased *srebp-1* transcript levels are due to reduced tissue 22:6n-3 composition, it may also be an overall response to the impaired LC-PUFA biosynthetic pathway. Taken together, our findings suggest that the expression levels of *srebp-1*, at least in the liver and pyloric caeca, is regulated by both the status of endogenous LC-PUFA synthesis and by dietary LC-PUFA levels. On the other hand, CRISPR/Cas9-induced mutations in Δ6abc/5^Mt^ had no major effect on the expression of *srebp-2* while expression level was downregulated in Δ6bc^Mt^ when fed low LC-PUFA diet. The reason for this reduced expression of *srebp-2* in Δ6bc^Mt^ is unclear. Notably, WTs fed low LC-PUFA diet demonstrated significant upregulation of *srebp-2* in the liver compared to WTs fed high LC-PUFA diet. The low LC-PUFA diet used in our study was partly formulated with plant oil which is known to contain phytosterols^40^ that reduce absorption of dietary cholesterol^41^. This probably induced *de novo* synthesis of cholesterol shown as an upregulation of *srebp-2*, a major transcription regulator of cholesterol biosynthesis in Atlantic salmon^42^. This observation is consistent with findings from functional genomics studies in Atlantic salmon^42^.

In conclusion, our study points to 20:4n-3 and 18:3n-3/18:2n-6 as the main *in vivo* substrate of salmon Δ5 Fads2 and Δ6 Fads2 respectively, and that the salmon Δ6 Fads2 possess Δ8-desaturation activities towards 20:3n-3 and 20:2n-6 *in vivo*. Our data also suggest Srebp-1 as a transcription regulator of salmon LC-PUFA biosynthesis and further show a combined effect of endogenous LC-PUFA synthesis and dietary LC-PUFA levels on the expression of *srebp-1*.

## Materials and Methods

### Cloning of target sequences for gRNAs

CRISPR-target sites were selected using a custom-made Perl script and publicly available genomic and cDNA sequence data for the Atlantic salmon genes *Δ6fads2-a*, *Δ6fads2-b*, *Δ6fads2-c* and *Δ5fads2* (Accessions: XM_014170212.1, NM_001172281.1, XM_014170389.1 and XM_014170354.1). As salmon *fads2* genes have similar coding exon structure with high degree of sequence homology^17^, a single CRISPR-target site was selected for simultaneous CRISPR-mediated edition of *Δ6fads2-a*, *Δ6fads2-b*, *Δ6fads2-c* and *Δ5fads2* or *Δ6fads2-b* and *Δ6fads2-c*. Candidate target sequences were screened against the current salmon genome assembly (GCA000233375.4) to avoid off-target genome editing events. For easy visual recognition of CRISPR-mutated salmon, *slc45a2* involved in melanin synthesis was mutated simultaneously with the *fads2* genes. Mutagenesis in *slc45a2* provided a suitable visual tracer that helps to screen WT from mutated fish, as CRISPR-induced mutations in both *slc45a2* and interested target genes in Atlantic salmon were highly correlated^34,35^, with fish displaying albino phenotypes peculiar to *slc45a2* in addition to phenotypes specific for target genes. Thus, mutations in *slc45a2* do not seem to influence rate of mutagenesis or phenotypes for the target genes. Candidate *fads2* target sequences and oligonucleotides used for cloning target sites are listed in Supplemental Table 3. To obtain double-stranded DNA inserts for *fads2* target sequences, one μg of each forward and reverse oligonucleotide was annealed in T4 ligase buffer (NEB) by incubating at 85 °C for 10 min and then cooling to room temperature. One μl of annealed oligonucleotide diluted 1:10 was ligated into 50 ng of the BsmBI-linearized pT7-gRNA (Addgene ID# 46759)^32^ plasmid using T4 DNA ligase (NEB) and transformed into competent DH5α cells. Recombinant plasmids were isolated using QIAprep Spin Miniprep kit (Qiagen).

### *In vitro* synthesis of gRNA and Cas9 mRNA

For producing gRNAs for the *fads2* genes, the respective pT7-gRNA plasmids were digested with BamHI-HF^TM^ (NEB) and purified using the DNA Clean and Concentrator^TM^-5 (ZYMO RESEARCH). The gRNAs were synthesized using the MEGAscript T7 kit (Ambion). Synthesized gRNAs were purified using the mirVana and miRNA Isolation kit (Ambion). The gRNA for *slc45a2* was prepared as previously described^33^. For making the Cas9 nuclease mRNA, the pTST3-nCas9n vector, codon optimized for zebrafish (Addgene ID# 46757)^32^ was digested with XbaI (NEB) and gel-purified using Wizard® SV Gel and PCR clean-up system (Promega). Cas9 mRNA was *in vitro* transcribed using the mMessage mMachine T3 kit (Ambion) and purified using RNeasy Mini Kit Spin column (Qiagen). The integrity of synthesized gRNAs and Cas9 mRNA was checked using the RNA 6000 Nano Kit and Agilent 2100 Bioanalyzer (Agilent Technologies).

### Microinjection

Atlantic salmon sperm and eggs were provided by Aquagen (Trondheim, Norway). Salmon eggs were fertilized with sperm in freshwater supplemented with 0.5 mM reduced glutathione at 6–8 °C^43^ and incubated at 6–8 °C for 2–3 h. Embryos were microinjected with 150 ng/μl of Cas9 mRNA and a mixture of two gRNAs each 50 ng/μl in Hepes buffer using the picospritzer III (Parker Automation). One of the gRNAs targets *slc45a2* and the other simultaneously targets *Δ6fads2-a*, *Δ6fads2-b*, *Δ6fads2-c* and *Δ5fads2* or *Δ6fads2-b* and *Δ6fads2-c*. Uninjected fertilized eggs were kept and used as wildtype controls. Microinjected and uninjected eggs were kept in freshwater at 6–8 °C until hatching. Shortly after feeding was started, fully albino juveniles were sorted, fin clipped or fully sampled to confirm CRISPR-induced mutations in the target genes. Samples were stored in 96% ethanol.

### Detection of CRISPR/Cas9-induced mutations

Extraction of genomic DNA from fin clips and tissues of salmon was performed using DNeasy Blood and Tissue Kit (Qiagen). Genomic Regions flanking the CRISPR-targets were PCR-amplified using DyNAzyme II DNA Polymerase (Thermo Scientific). Sequences of PCR primers are listed in Supplemental Table 4. Using BigDye™ Terminator v3.1 cycle sequencing kit (Applied Biosystems™), gel-purified direct PCR fragments and subcloned PCR products in the pCR4 – TOPO® vector (Invitrogen) were sequenced. DNA sequencing chromatograms were analyzed using the Unipro UGENE^44^.

### Feeding trial

The feeding trial was carried out at the Institute of Marine Research (Matre, Norway) from 15^th^ January 2018 to 9^th^ of March 2018. Shortly after hatching, Δ6abc/5^Mt^, Δ6bc^Mt^ and wildtypes (WTs) were fed 227 days with standard commercial diet (Nutra Olympic, Skretting Nutreco Company) which provides enough of LC-PUFAs (Supplemental Table 2). Notably, after feeding with standard diet, we had no WT with exact same size as the CRISPR-mutated fish even though WT were obtained from the same batches of eggs as the CRISPR-mutated fish. As the CRISPR-mutated fish and WT were in separate tanks, we suspected that size differences between fish may be due to tank variation effects, most likely resulting from differences in density of fish per tank (which can influence amount of feed given) as well as size and type of tanks. On the other hand, the Δ6bc^Mt^ and Δ6abc/5^Mt^ were observed to be similar in size with estimated average weights of 49 g compared to WT with estimated average weights of 85 g, and so we cannot rule out impact of *fads2* gene knockout on growth. Consequently, all three groups of experimental fish were fed 110 days with diets containing higher levels of 22:6n-3, DHA-1 and DHA-2 (SPAROS, Portugal), (Supplemental Table 2) until an approximate average weight of 85 ± 25 g for Δ6abc/5^Mt^, 104 ± 25 g Δ6bc^Mt^ and 176 ± 34 g WTs (Table 1). Due to the probable tank variation effects, we decided to use a “common garden” experimental setup, where all three fish groups were in the same tanks during feeding with low LC-PUFA or high LC-PUFA diet (SPAROS, Portugal) and also with DHA-rich diets. For the low LC-PUFA and high LC-PUFA feeding trial, six tanks, each containing a total of 18 fish comprising of 6 each of Δ6abc/5^Mt^, Δ6bc^Mt^ (Pit-tagged) and WTs were set up. Pit-tags were intended to differentiate Δ6bc^Mt^ from Δ6abc/5^Mt^ as both had albino phenotypes. Δ6bc^Mt^ were identified from Δ6abc/5^Mt^ using ARE-H5 portable reader (TracID Systems Company). Three tanks were fed a low LC-PUFA diet with reduced 20:5n-3 and 22:6n-3 content but higher levels of 18:3n-3 and 18:2n-6 (Supplemental Table 2) while the other 3 tanks were fed a high LC-PUFA diet rich in 20:5n-3 and 22:6n-3 but reduced composition of 18:3n-3 and 18:2n-6 (Supplemental Table 2) for 54 days. The feed was supplied continuously and in excess using automatic feeders (Arvotec single feeder). The freshwater temperature was 7.7–12.5 °C with at least 70 ppm oxygen saturation at the outlet. The average weights and lengths of Δ6abc/5^Mt^, Δ6bc^Mt^ and WTs after the feeding with standard diet, 22:6n-3 (DHA)-rich diets and low and high LC-PUFA diets are summarized in Table 1. Tissues from 6 fish each of Δ6abc/5^Mt^, Δ6bc^Mt^ and WTs per diet were sampled after the 54 days of feeding. White muscle and liver were quickly frozen on dry ice and stored at −80 °C until analysis. Tissues of pyloric caeca were first immersed in RNALater (Thermo Fisher Scientific) and later stored at −80 °C.

### Lipid extraction and GC-MS analysis

Total lipids were extracted from approximately 300 mg of white muscle and liver tissues from three fish per dietary treatment according to Folch *et al*.^45^. The lipid content per tissue was determined gravimetrically. Phospholipids and triacylglycerols (TAG) were separated by high-performance thin-layer chromatography (HPTLC) silica gel 60 plates (10 × 10 cm, Merck) using hexane/diethyl ether/acetic acid (80:20:2, v/v) as developing solvent^46^. Lipid classes were visualized through brief exposure to iodine vapor. Lipid classes were scraped off and fatty acid methyl esters (FAMEs) produced by acid-catalyzed transesterification performed at 50 °C overnight^47^. FAMEs were identified and quantified using gas chromatography (Agilent 7890) equipped with mass spectrometer (Agilent 5977B) using 25 m × 0.25 mm capillary column (CP-Wax 52CB, Agilent). Helium was used as carrier gas and temperature programming was from 90 °C to 150 °C at 30 °C/min and then to 230 °C at 2.5 °C/min and finally to 240 °C at 10 °C/min and held for 23 min.

### Preparation of hepatocytes and incubation with ^14^C-18:3n-3 for assay of fatty acyl desaturation/elongation activities

Hepatocytes from three each of Δ6abc/5^Mt^ and WT salmon fed low LC-PUFA diet were prepared as previously described^48^ with modifications. Liver was dissected, quickly perfused through hepatic vein, finely chopped and incubated for 45 mins at 20 °C in 20 ml of solution A (Hank’s balanced salt solution with 10 mM Hepes and 1 mM EDTA) containing 1 mg/ml collagenase (Sigma). Digested liver tissues were filtered through 100 μm cell strainer (Sigma) and the cells collected by centrifugation at 400 × *g* for 3 min. The cell pellets were washed with 20 ml of solution A containing 10 mg/ml fatty acid free bovine serum albumin (FAF-BSA, Sigma) and centrifuged at 400 × *g* for 3 min. Cell pellets were further washed and resuspended in 5 ml of solution B (calcium free minimum essential medium containing 100 U/ml Penicillin, 100 μg/ml Streptomycin and 0.25 μg/ml Amphotericin B). For each liver sample, 1.904 ml of hepatocytes and 96 μl of ^14^C-18:3n-3 with approximate final concentration of 4.55 μM (0.5 μCi) was incubated for 2 hours at 20 °C. Cells were thereafter isolated by centrifugation at 400 × *g* for 2 min and washed with 2 ml solution B containing 10 mg/ml FAF-BSA. Total lipids were extracted as described by Folch *et al*.^45^. Transmethylation was performed by adding 1 ml toluene and 2.5 ml 1% (v/v) H_2_SO_4_ in methanol and incubating at 50 °C overnight. FAMEs were extracted by adding 2 ml 2% (w/v) KHCO_3_ and 5 ml hexane/diethyl ether (1:1, v/v) containing 0.01% (w/v) butylated hydroxyl toluene (BHT) and then centrifuging at 2879 × *g* for 5 min. FAMEs in the upper phase were dried under a stream of nitrogen and resuspended in 100 μl hexane containing 0.01% BHT. FAMEs were applied as 2 cm streaks on a 20 × 20 silica gel TLC plate (Sigma-Aldrich) pre-coated with 0.1 g/ml silver nitrate in acetonitrile. The plate was developed in toluene/acetonitrile (95:5, v/v) and then desiccated in the dark for 30 min. Autoradiography was performed by placing the plate together with Kodak BioMax MR2 film in an autoradiography exposure cassette for 6 days at room temperature and then developed in Carestream Kodak GBX Developer and Carestream Kodak GBX Fixer. Percentage radioactivity in n-3 PUFAs was determined by scraping corresponding bands into 1 ml of scintillation cocktail and then counted in a liquid scintillation analyser (TRI-CARB 2900TR, Packard).

### Tissue RNA isolation and gene expression analysis by RT-qPCR

Total RNA from liver and pyloric caeca was extracted using the RNeasy Plus Universal Mini kit (Qiagen) with genomic DNA elimination buffer according to the manufacturer’s protocol. Using the Agilent RNA 6000 Nano kit and an Agilent 2100 Bioanalyzer (Agilent Technologies), the integrity of isolated RNA was checked with an RNA integrity value range of 8.9–10 obtained for liver and 6.6–8.8 for pyloric caeca. One μg of total RNA was reverse transcribed using the QuantiTect® Reverse Transcription kit (Qiagen) according to the manufacturer’s protocol. A negative control with no reverse transcriptase was used to check for genomic DNA contamination. The mRNA expression of *Δ6fads2-a*, *Δ6fads2-b*, *Δ6fads2-c*, *Δ5fads2*, *srebp-1*, *srebp-2*, fatty acid synthase-a (*fas-a*) and *fas-b* was measured by RT-qPCR using LightCycler® 96 (Roche). The LinRegPCR analysis program^49,50^ was used to calculate PCR efficiencies and Ct-values from raw amplification data generated from the RT-qPCR. The fold change of gene expression between CRISPR-mutated fish and WTs or between WTs under the two dietary regimens was determined using the qBase relative quantification framework and software^51^. Fold changes of gene expression in the two individual strains, Δ6abc/5^Mt^ and Δ6bc^Mt^ were determined relative to the WTs fed either low LC-PUFA or high LC-PUFA diet in order to assess the impact of both CRISPR mutations and dietary treatment on gene expression. On the other hand, the effects of only dietary LC-PUFA levels on gene expression was measured by determining fold changes of gene expression in WTs fed low LC-PUFA diet relative to WTs under high LC-PUFA diet. Elongation factor 1 alpha- b (*ef1α-b*) pre-validated in Atlantic salmon^52^ was used as a reference gene. All primers used in RT-qPCR are listed in Supplemental Table 5.

### Statistical analysis

Weights and lengths of fish fed low and high LC-PUFA diets were analyzed by two-way ANOVA using dietary treatment and strain or genotype as experimental factors, followed by multiple comparisons of the means using Tukey HSD post-hoc test. Weights and lengths of fish fed DHA-rich diets were analyzed by one-way ANOVA using strain as experimental factor, followed by Tukey HSD post-hoc test. Analyses were performed using GraphPad Prism 7 software. All fatty acid data are presented as means ± standard deviation with N = 3 unless otherwise stated. Statistical differences between CRISPR-mutated fish and WTs were determined by two-tailed t-test with unequal variance. All RT-qPCR data are presented as means ± confidence interval (N = 5). Differences in gene expression between CRISPR-mutated fish and WTs or between WTs under the two dietary regimens were determined by Mann-Whitney test with two-sided significance.

### Ethics statement

All experiments on animals were performed in strict accordance with the Norwegian Animal Welfare Act of 19^th^ of June 2009. Experiments carried out in this study were approved by the Norwegian Animal Research Authority (NARA 5741). Unnecessary pain was avoided by anaesthetizing all fish with Finquel MS-222 (Scan Vacc) prior to euthanizing and tissue sampling.

## Supplementary information

Supplemental data
