# Supplementary material for: CRISPR/Cas9-mediated editing of Δ5 and Δ6 desaturases impairs Δ8-desaturation and docosahexaenoic acid synthesis in Atlantic salmon (Salmo salar L.)
Source: Sci Rep. 2019 Nov 15;9:16888. doi: 10.1038/s41598-019-53316-w (PMC6858459; doi:10.1038/s41598-019-53316-w)
Supplement: Supplementary file 1 — Supplemental data [file 41598_2019_53316_MOESM1_ESM.pdf]

CRISPR/Cas9-mediated editing of Δ5 and Δ6 desaturases impairs Δ8-desaturation and docosahexaenoic acid synthesis in Atlantic salmon (*Salmo salar* L.)

Alex K. Datsomor [1], Rolf E. Olsen [1, 2], Nikola Zic [1], Angelico Madaro [2], Atle M. Bones [1], Rolf B. Edvardsen [2], Anna Wargelius [2], Per Winge [1*]

[1]Norwegian University of Science and Technology, Institute of Biology, Trondheim, 7491, Norway

[2]Institute of Marine Research, Bergen, NO-5817, Norway

*Corresponding author: per.winge@ntnu.no

Alex K. Datsomor: alex.datsomor@ntnu.no

Rolf E. Olsen: rolf.e.olsen@ntnu.no

Nikola Zic: nikolaz@stud.ntnu.no

Angelico Madaro: angelico.madaro@hi.no

Atle M. Bones: atle.m.bones@ntnu.no

Rolf B. Evardsen: rolf.brudvik.edvardsen@hi.no

Anna Wargelius: anna.wargelius@hi.no

Per Winge: per.winge@ntnu.no

	**Total number of albinos screened**	**Number of fish with CRISPR-indels in Δ6 & Δ5 *fads2* genes**	**Number of albinos with unedited *fads2* gene**
Δ6abc/5^{Mt}	56	56	0
Δ6bc^{Mt}	55	53	2

Supplemental table 1. Correlation between albinos and CRISPR-induced indels in Atlantic salmon *fads2* genes

	**% of total fatty acids**				
	**Standard diet**	**DHA-1**	**DHA-2**	**Low LC-PUFA**	**High LC-PUFA**
**Saturates**					
C14:0	4.98	5.93	6.21	3.74	7.69
C16:0	14.99	21.78	20.31	11.75	17.82
C18:0	3.06	3.82	3.80	3.18	3.15
**Monoenes**					
C16:1n7	5.46	4.97	5.84	2.90	7.43
C18:1n7	2.66	3.45	3.70	2.56	3.45
C18:1n9	22.14	14.32	13.95	23.93	12.54
C22:1n9	0.37	0.00	0.00	0.14	0.25
**n-6 fatty acids**					
C18:2n6	7.56	9.78	10.34	20.69	8.69
C20:2n6	0.17	0.19	0.20	0.08	0.15
C20:4n6	0.74	0.83	0.91	1.04	0.74
**n-3 fatty acids**					
C18:3n3	3.11	1.36	1.54	16.36	2.04
C20:3n3	0.09	0.13	0.13	0.05	0.11
C20:4n3	0.56	0.45	0.56	0.06	0.47
C20:5n3	9.05	7.61	9.28	1.63	10.10
C22:5n3	1.37	1.16	1.33	0.17	1.29
C22:6n3	7.90	12.44	10.98	2.13	8.64

Supplemental table 2. Fatty acid composition (% of total fatty acids) in the standard commercial diet, the two diets relatively rich in docosahexaenoic acid (22:6n-3, DHA), namely DHA-1 and DHA-2, and low LC-PUFA and high LC-PUFA diets

Name	Sequence (5' - 3')	Targeted gene(s)
Δ6abc/5^{Mt} target	GGCACCGACAGAGCCCAGCCAGG**	*Δ6fads2-a, Δ6fads2-b, Δ6fads2-c, Δ5fads2*
Δ6bc^{Mt} target	**CCAAGGGTGGCGTGGTTGGGCCC	*Δ6fads2-b, Δ6fads2-c*
slc45a2 target	GGGGAACAGGCCGATAAGACTGG**	*slc45a2*
Δ6abc/5-F	TAGGCACCGACAGAGCCCAGCC	
Δ6abc/5-R	AAACGGCTGGGCTCTGTCGGTG	
Δ6bc-F	TAGGGCCCAACCACGCCACCCT	
Δ6bc-R	AAACAGGGTGGCGTGGTTGGGC	
slc45a2-F	TAGGGGAACAGGCCGATAAGAC	
slc45a2-R	AAACGTCTTATCGGCCTGTTCC	

Supplemental table 3. CRISPR-target sequences and oligonucleotides, **Underlined trinucleotides are the CRISPR protospacer adjacent motif (PAM) sites: -F and -R indicate the respective forward and reverse oligonucleotide.

Primer name	Sequence (5' – 3')	Target gene(s) amplified
Δ6abc/5-1F	CGGTAAACCCCTGTTAGTAGAT	*Δ6fads2-a*
Δ6abc/5-1R	AGACGCTCTAGGCTTCACATTC	
Δ6abc/5-2F	GTTAGTGGATTGTACATTACTGG	*Δ5fads2*
Δ6abc/5-2R	ATATCTCCATCCACATCATACCT	
Δ6abc/5-3F	TTTACTGAGAGGACATTTGTATG	*Δ6fads2-c*
Δ6abc/5-3R	TGTCTGAGAAGCATGGAGAAAG	
Δ6abc/5-4F	AACAACCAGGAAATGGCAGAGC	*Δ6fads2-b*
Δ6abc/5-4R	AGAGACGCTCTAGGCTTCACAT	
Δ6bc-1F	ACAGTCACAGTCATCAAATCAG	*Δ6fads2-b* and *Δ6fads2-c*
Δ6bc-1R	TTCCTGTCGATGACCAACCACT	

Supplemental table 4. Primers used in CRISPR-target site PCR amplification

Primer	Forward primer 5'-3'	Reverse primer 5'-3'	Accession #**
srebp-1	GCCATGCGCAGGTTGTTTCTTCA	TCTGGCCAGGACGCATCTCACACT	HM561860.1
Δ5fads2	GTGAATGGGGATCCATAGCA	AAACGAACGGACAACCAGA	AF478472.3
Δ6fads2-a	CCCCAGACGTTTGTGTCAG	CCTGGATTGTTGCTTTGGAT	AY458652.3
Δ6fads2-b	CAGTCTGGTTGTCCGTTCGT	GATATTTCACTGGCTGTGTCC	NM_001172281.1
Δ6fads2-c	CCTGCGAACAGAGATAGATTGA	GATTTACGCTTGAGCTAACATC	GU207401.1
fas-a	GTATGGCACTACCCAAATACTG	AGGTACGAGCTGAATCTATCCA	XM_014179803.1
fas-b	GGTAGTGCCATACAAGTGATGT	TCAGTGGGCACCAAACATGAAC	XM_014209529.1
elf1α-b	TGCCCCTCCAGGATGTCTAC	CACGGCCCACAGGTACTG	BG933853.1
srebp-2	TCGCGGCCTCCTGATGATT	AGGGCTAGGTGACTGTTCTGG	NM_001195819.1

Supplemental table 5. List of primers used for RT-qPCR, ** GenBank (http://www.ncbi.nlm.nih.gov/)

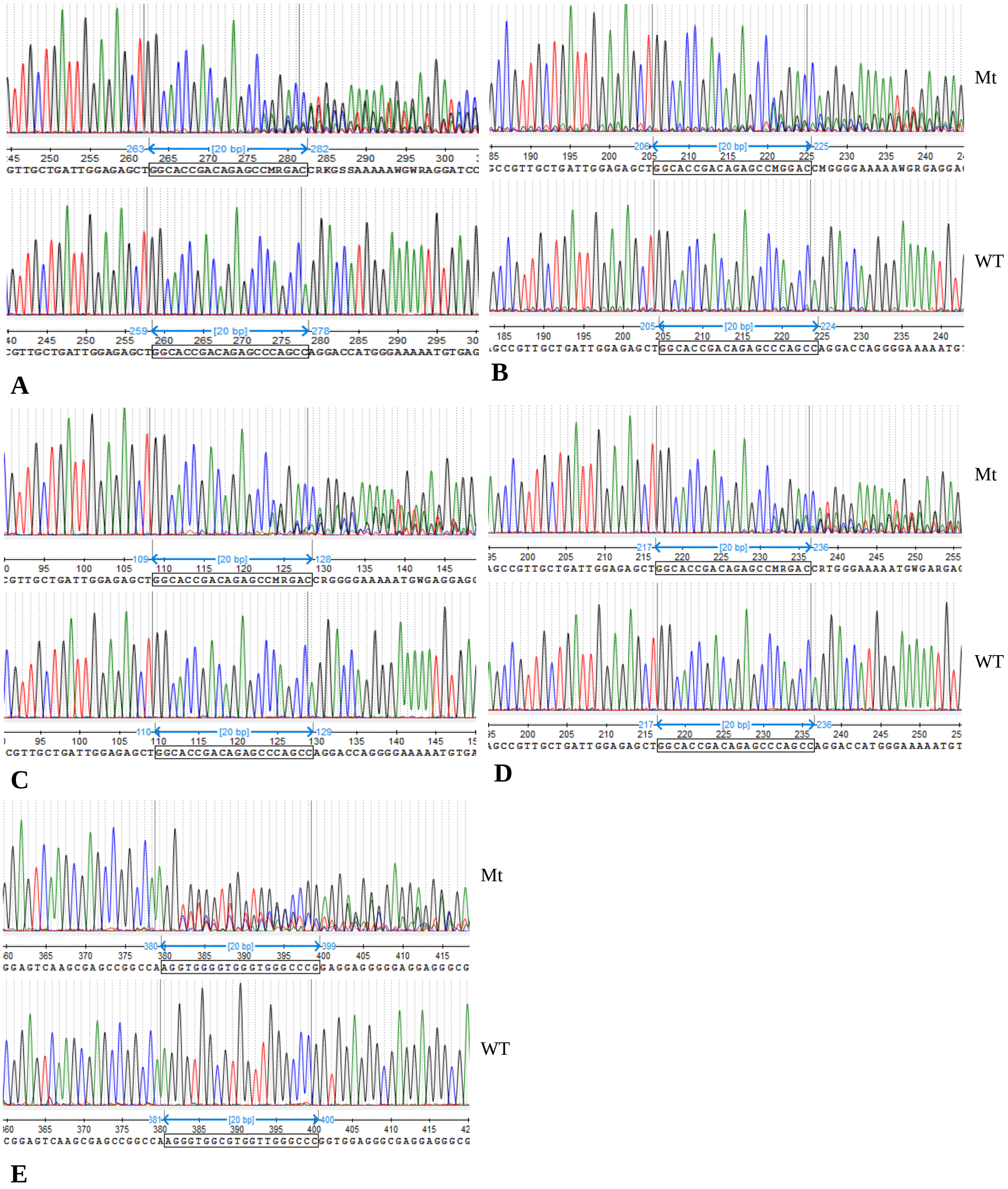

Supplemental figure 1. Confirmation of CRISPR/Cas9-induced mutations in *Δ6fads2-a* (A), *Δ6fads2-b* (B), *Δ6fads2-c* (C) and *Δ5fads2* (D) from a $\Delta6abc/5^{Mt}$ individual, and in *Δ6fads2-b* and *Δ6fads2-c* (E) from $\Delta6bc^{Mt}$, shown as scrambled peaks at CRISPR-target sites in DNA sequencing chromatograms obtained by direct sequencing of gel-purified PCR products flanking regions around the target sites. CRISPR-target sequences are highlighted in boxes below the DNA sequencing chromatograms. CRISPR-target site in *Δ6fads2-b* and *Δ6fads2-c* of $\Delta6bc^{Mt}$ were co-amplified due to high level of sequence homology between *Δ6fads2-b* and *Δ6fads2-c*. Mutant DNA chromatograms from CRISPR-mutated fish (Mt) are compared to that of wildtypes (WT) that were used as controls in the experiments. Chromatograms were analyzed using the Unipro UGENE [44].

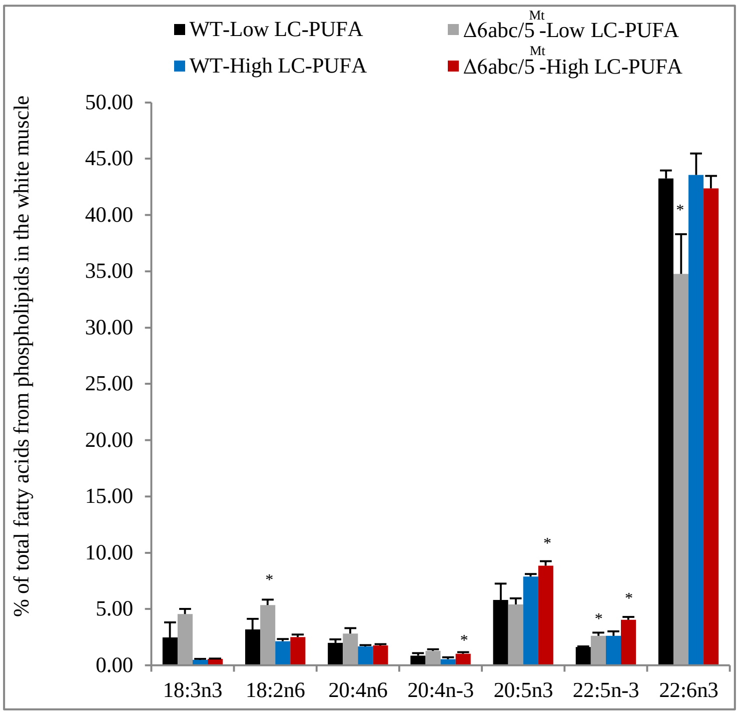

Supplemental figure 2. Polyunsaturated fatty acid composition of phospholipid pool in the white muscle of wildtypes (WT) and Δ6abc/5^{Mt} salmon fed low LC-PUFA and high LC-PUFA diets for 54 days. Phospholipids were separated on high performance thin layer chromatography silica gel 60 plates. Fatty acid methyl esters (FAMEs) were prepared by acid-catalyzed transesterification and quantified by gas chromatography coupled with mass spectroscopy. Results are shown as mean ± standard deviation of 3 fish. Statistical differences between WT and CRISPR-mutated fish were determined using two-tailed t-test with unequal variance and are denoted as asterisks (*$p \leq 0.05$).

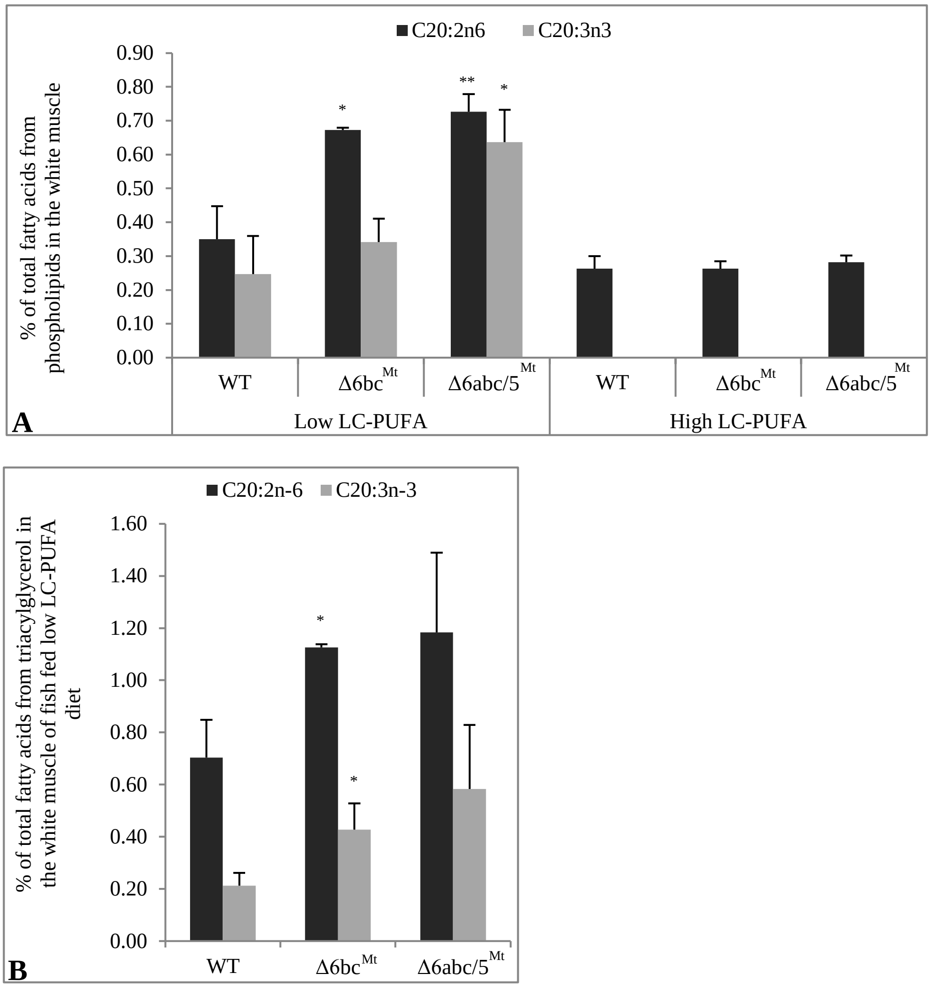

Supplemental figure 3. White muscle 20:2n-6 and 20:3n-3 composition in phospholipids (A) and triacylglycerol (B) in wildtypes (WT), $\Delta6bc^{Mt}$ and $\Delta6abc/5^{Mt}$ fed low LC-PUFA and high LC-PUFA diets for 54 days. The composition of 20:3n-3 in white muscle phospholipids of fish fed high LC-PUFA diet was very low and could not be measured in all biological replicates. Phospholipids and triacylglycerol were separated on high performance thin layer chromatography silica gel 60 plates. Fatty acid methyl esters (FAMEs) were prepared by acid-catalyzed transesterification and quantified by gas chromatography coupled with mass spectroscopy. Results are shown as mean ± standard deviation of 3 fishes except samples from $\Delta6bc^{Mt}$ with N = 2. Statistical differences between WTs and CRISPR-mutated fish were determined using two-tailed t-test with unequal variance and are denoted as asterisks (*$p \leq 0.05$, **$p < 0.01$).

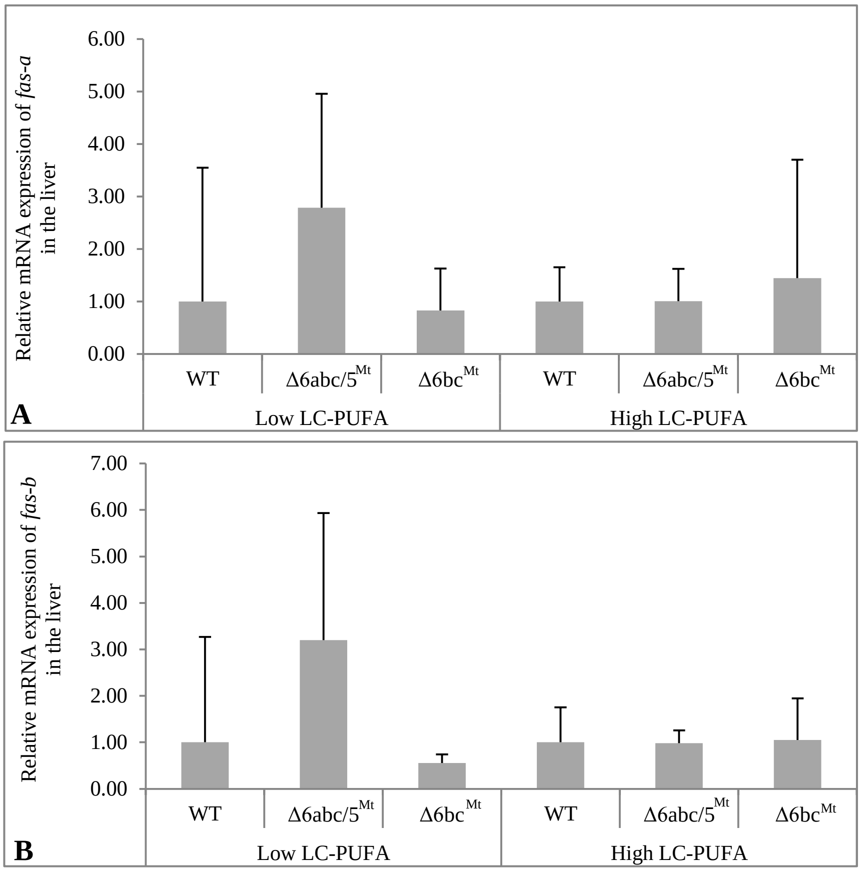

Supplemental figure 4. The expression of liver fatty acid synthase-a, *fas-a* (A) and *fas-b* (B) in $\Delta6abc/5^{Mt}$ and $\Delta6bc^{Mt}$ relative to wildtypes (WT) fed low LC-PUFA and high LC-PUFA diets for 54 days. Results are presented as mRNA expression in CRISPR-mutated fish relative to WT, with WT set to 1. All qPCR data were analyzed using qBase^{+} [51] which determined statistical differences between WT and CRISPR-mutated fish using unpaired Mann-Whitney test with two-sided significance. Data are presented as means ± confidence interval with N = 5 per dietary treatment. Normalization was performed using elongation factor 1α-b (*ef1α-b*).
